# The Sex Features of Patients With Solid Pseudopapillary Neoplasms of the Pancreas: A Retrospective Study

**DOI:** 10.3389/fonc.2022.844182

**Published:** 2022-02-18

**Authors:** Guangmin Wei, Qiong Luo, Jiankai Fang, Xiaolou Li, Yanhong Shi, Yuqiong Li, Liqi Sun

**Affiliations:** ^1^ Department of Oncology, Mengchao Hepatobiliary Hospital of Fujian Medical University, Fuzhou, China; ^2^ Department of Gastroenterology, Changhai Hospital, Second Military Medical University, Shanghai, China; ^3^ Department of Gastroenterology, 72^nd^ Group Army Hospital, Huzhou University, Huzhou, China

**Keywords:** pancreas, solid pseudopapillary tumor, survival, risk factors, retrospective study

## Abstract

**Background:**

Solid pseudopapillary neoplasms of the pancreas (SPNs) in male patients are more frequently reported. The aim of the study was to evaluate the sex features of SPN and the risk factors that predict tumor recurrence.

**Methods:**

From 2013 to 2019, patients who were pathologically confirmed to have SPNs were retrospectively reviewed. The baseline study parameters were compared between males and females. A logistic regression model was established to identify the independent risk factors for tumor recurrence.

**Results:**

In total, 221 patients were included in this study. Of them, 53 patients (24.0%) were males. Male patients were older than female patients (39.1 vs 31.6 years, P=0.001), and the tumor size in male patients was smaller than that in female patients (50.38 vs 39.65 mm, P=0.038). The preoperative imaging diagnostic accuracy was significantly higher in females than in males (70.5% vs 54%, P=0.02). SPNs in male patients tended to be misdiagnosed with other malignant tumors (37.7% vs 10.7%, P<0.0001), with a more solid component observed in images (66.8% vs 24.7%, P<0.0001). For immunohistochemical staining, the expression of beta catenin was significantly lower in male patients (P=0.002), and the expression of vimentin was the opposite (P=0.01). The overall survival rate and disease-free survival were not different. Based on multivariate analysis, older age [hazard ratio (HR)= 1.094, 95% confidence interval (CI): 1.005-1.190] and KI 67 index grade III (HR=12.029, 95% CI: 2.399-60.311) were independent risk factors for tumor recurrence.

**Conclusion:**

The clinical and imaging features of SPN in males were not in full accord with those in females; however, the differences did not influence prognosis.

## Introduction

Solid pseudopapillary neoplasms (SPNs) are uncommon. These tumors account for approximately 0.9%-2.7% of all exocrine pancreatic neoplasms (1, 2) and approximately 3%-5% of pancreatic cystic neoplasms (3, 4). The tumor is an epithelial-originated low-grade malignant neoplasm with the possibility of locally advanced, recurrent, and metastatic disease. Complete surgical resection is recommended as the main treatment for SPN (5).

SPNs occur predominantly in young women, with an overall female-male ratio of 9.8:1 reported in previous studies (1, 2). However, more male patients were identified in recent studies, with a female-male ratio ranging from 5:1 to 3:1 (6–9). The phenomenon that more male SPN patients have been identified has attracted increasing attention from clinicians and researchers (10).

Previous studies identified that progesterone receptors were present in 79%-100% of cases with SPN (11, 12). Moreover, the imaging features were different between male and female patients (13–15). In general, we hypothesized that progesterone may play a role in the biological behavior and clinical characteristics of SPN, which needs to be further clarified. However, a limited number of studies have focused on the impact of sex on the prognosis of patients with SPN. The conclusion was inconsistent (2, 7, 16, 17). The sample size in most of these studies was small, and the study cohort in one study was extracted from a database with nonstandard data included (7). Therefore, this study aims to compare the clinicopathological features and prognosis between males and females with SPN based on a relatively large cohort study from 2 pancreatic disease centers in China.

## Methods

### Study Population

**Figure 1:**
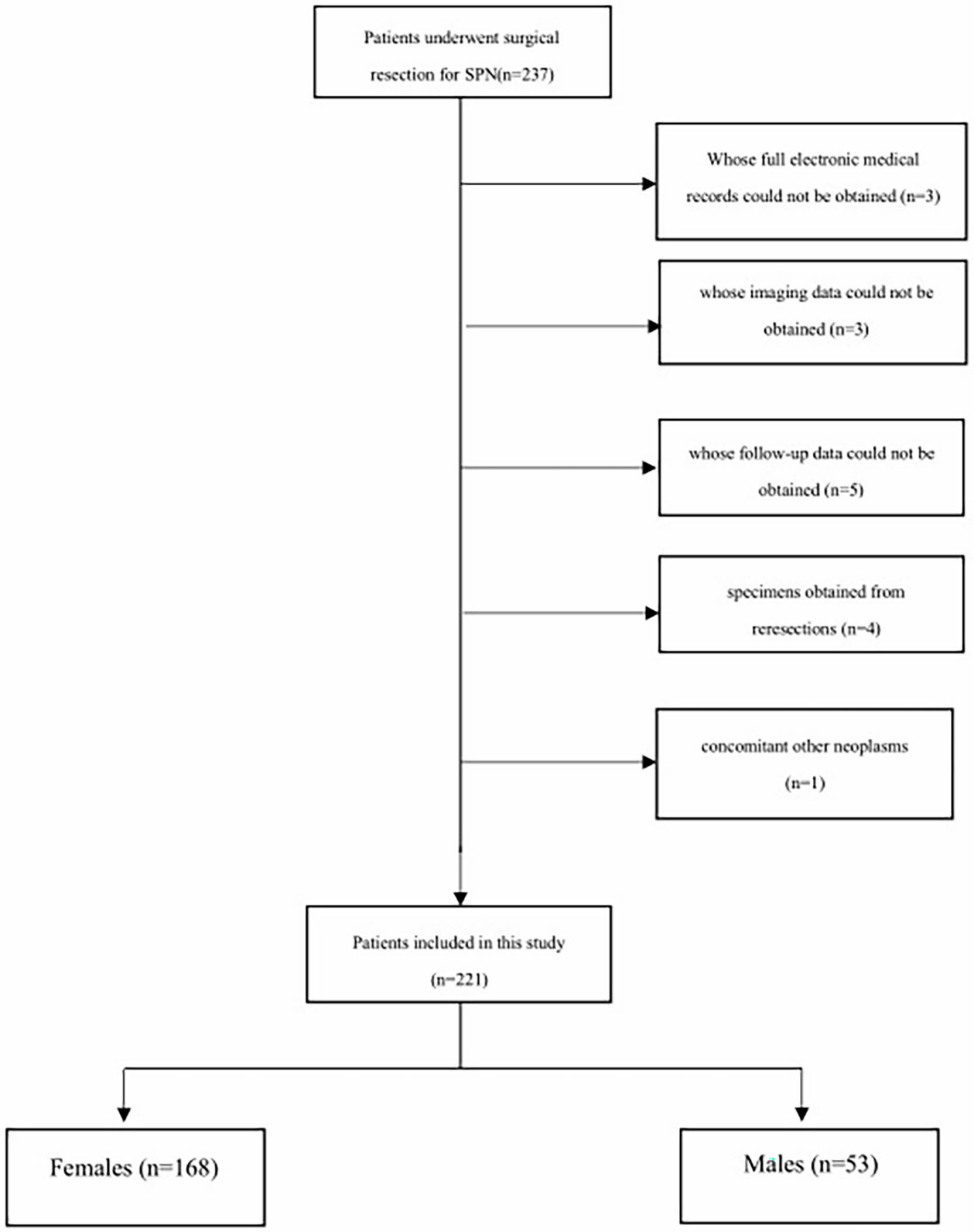
Flowchart presenting the selection process of studies.

We conducted a retrospective study at Changhai Hospital affiliated with Navy/Second Medical University. The Institutional Review Board approved the study. Patients who underwent surgical resection from January 2013 to June 2019 for pathologically identified SPN were included in our study. The following inclusion criteria were applied: (1) patients pathologically diagnosed with SPN; (2) patients whose full electronic medical records and imaging records could be obtained; and (3) patients whose follow-up data could be obtained. Exclusion criteria included (1) specimens obtained from reresections; (2) concomitant other neoplasms on final pathology (e.g., neuroendocrine tumor, cholangiocarcinoma); and (3) patients with unavailable pathological and follow-up data. The selection procedure of the study participants is presented in **Figure 1**.

The decision on surgical treatment was made by multidisciplinary hepatopancreatobiliary teams. If the lesion was diagnosed as a pancreatic cystic lesion by imaging modality, the surgical indications followed the International Consensus Guideline (18). If diagnosed with invasive cancer, the surgery indications would follow the European Society for Medical Oncology guidelines (19). The choices of surgical procedures depended on the location, degree, extent of diseases and experiences of the surgeon.

### Perioperative Management

The operations for SPN were performed by experienced surgeons in Changhai hospital. Before surgery, routine preoperative examinations were performed to exclude surgical contraindications. These routine examinations included electrocardiogram, pulmonary function, coagulation function, liver function, renal function, electrolyte and hemoglobin.

After surgery, amylase analysis from drainage fluid was performed to determine whether postoperative pancreatic fistula (POPF) existed. Routine blood examinations were performed to determine whether infection existed and whether antibiotics were used. Plain CT was performed to determine whether pancreatic fluid collection existed and to detect the causes of infection. If any clinically significant complications occurred, further treatments were needed.

### Study Parameters

The parameters included in our study were composed of five parts: baseline characteristics, imaging data, pathological outcomes, short-term complications and long-term follow-up data.

The baseline characteristics included age, sex, initial symptoms, hospital stay days and surgical methods. The surgical methods included pancreatoduodenectomy (PD), total pancreatectomy (TP), distal pancreatectomy (DP), central pancreatectomy (CP) and enucleation.

The imaging data included tumor size, location, imaging diagnoses by radiologists and proportion of solid components. The tumor might be located in head/body/tail/multiple sites of the pancreas. If multiple tumors occurred, only the size of the largest tumor was measured. The proportion of solid components was evaluated by T2 sequences obtained by contrast-enhanced MRI. If the proportion of the solid component was inconsistent on different layers, only the largest proportion was measured. The imaging diagnosis was divided into 2 parts: diagnostic accuracy (the diagnostic conclusions were SPNs) and surgical indication accuracy [diagnostic conclusions were malignancy, SPNs, neuroendocrine tumor (NET)].

The pathological outcomes included margin status, peripheral tissue invasion status and immunohistochemical outcomes. Positive margin status was defined as a tumor component ≤5 mm from the incisal margin. Peripheral tissue invasion status consisted of perineural invasion, vascular invasion, cancerization of ducts, lymphatic metastasis, common bile duct invasion, peripancreatic fat invasion, spleen invasion and duodenum invasion. The immunohistochemical outcomes included the Ki-67 index, beta-catenin, lymphoid enhancer-binding factor (LEF1), cyclin D1 and vimentin, which were measured in all included cases. The Ki-67 index of the tumor was divided into 3 grades: < 3%, 3%-20% and >20% (20).

Short-term complications were adverse events that occurred within 30 days after surgery, including POPF, delayed gastric emptying, hemorrhage, abdominal infection and bile leakage. The grade of complications was based on the Clavien-Dindo score. Complications that scored Clavien Dindo grade III or greater were considered severe complications.

The long-term follow-up data included long-term complications (exocrine insufficiency, endocrine insufficiency, alimentary stricture), disease-free survival (DFS) rate and overall survival (OS) rate. Follow-up data were obtained from telephone interviews and/or outpatient interviews in this study. Endocrine insufficiency was defined as a fasting plasma glucose level > 7.0 mmol/L and/or the need for diet modification, oral medication, or insulin use to control plasma glucose levels. Exocrine insufficiency was defined as symptoms (steatorrhea or weight loss) resolving after pancreatic enzyme supplementation (21). Recurrence was defined as a local or a metastatic tumor confirmed by radiology or histology during postoperative follow-up.

### Statistical Analysis

The patients were divided into 2 groups according to their sex: male group and female group. The parameters were compared between the 2 groups. Quantitative parameters were expressed as the medians and range. Continuous data are reported as the mean ± standard deviation (SD) or as the median and range according to the distribution of the parameter. Categorical parameters were compared between the 2 groups using χ^2^ or Fisher’s exact test. Kaplan–Meier survival curves were established to estimate and compare the RFS rate and OS rate between the 2 groups. A logistic regression model was established to identify the independent risk factors for tumor recurrence. Statistical analysis was performed using SPSS version 22.0 (SPSS Inc., Chicago, IL, United States), and survival curves were drawn using GraphPad Prism (version 7.00). All tests were two-sided, and a P value < 0.05 was considered statistically significant.

## Results

### Patient Characteristics

From January 2013 to December 2018, 221 patients who underwent pancreatic surgery were included in our study. All tumors were confirmed as SPNs according to the final histology examination. Among them, 53 patients (25.1%) were males, and 168 patients (74.9%) were females. The mean age of the overall study cohort was 33.3 years ± 12.7. Male patients were older than female patients (39.1 vs 31.6 years, t=3.283, P=0.001).

The majority of them were incidentally found (158/221, 71.5%). In the patients who were symptomatic, abdominal pain was the most common symptom (40/63, 63.5%), followed by abdominal distension (12/63, 19.0%), nausea and vomiting (8/63, 12.7%) and jaundice (3/63, 4.8%). The proportion of initial symptoms was not significantly different between males and females (χ^2 =^ 5.37, P=0.251).

In male patients, PD was performed in 13 patients (24.5%), DP was performed in 25 patients (47.2%), CP was performed in 10 patients (18.9%) and enucleation was performed in 5 patients (9.5%). No TP was performed in male patients. In female patients, PD was performed in 48 patients (28.6%), DP was performed in 82 patients (48.8%), CP was performed in 20 patients (11.9%), enucleation was performed in 8 patients (4.8%) and TP was performed in 10 patients (6.0%). The proportion of surgical methods was also not significantly different between males and females (χ^2 =^ 4.28, P=0.37).

The mean hospital stay was 12.53 days ± 6.87, with no difference between the 2 groups (14.24 vs 12.1 days, t=2.131, P=0.08).

The baseline characteristics of the study cohort are shown in **Table 1**.

**Table 1:** Baseline characteristics and imaging data stratified by gender.

	Male (N=53)	Female (N=168)	*P* value
**Age(years), mean ±SD**	39.1±12.7	31.6±12.2	0.001
**Symptoms, N%**			0.251
incidentally found	42(79.2)	116(69.0)	
abdominal pain	10(18.9)	30(17.9)	
abdominal distension	0(0)	12(7.1)	
nausea and vomiting	0(0)	8(4.8)	
jaundice	1(1.9)	2(1.2)	
**Surgical methods, N%**			0.370
PD	13(24.5)	48(28.6)	
DP	25(47.2)	82(48.8)	
CP	10(18.9)	28(16.7)	
TP	0(0)	10(6.0)	
enucleation	5(9.4)	8(4.8)	
**Hospital stays (days), mean ±SD**	14.2±9.3	12.0±6.0	0.08
**Tumor size (mm), mean ±SD**	39.6±22.8	50.4±28.7	0.038
**Tumor location (**head/body/tail/ diffuse**), N%**	23/5/4/21	67/19/19/63	0.92
**Imaging diagnoses, N%**			
SPN	28(54.0)	118(70.5)	0.02
Malignant tumor	20(37.7)	18(10.7)	<0.0001
neuroendocrine tumor	1(1.9)	11(6.5)	0.192
other benign tumors	4(7.5)	21(12.8)	0.321
**Imaging diagnoses for having surgical indications, N%**	49(92.5)	147(87.5)	0.519
**Solid component, N%**			
Completely solid	25(47.2)	40(23.8)	0.001
Completely cystic	0(0)	32(19.0)	<0.001
>50% solid component	23(43.4)	55(32.7)	0.157
<50% solid component	5(9.4)	41(24.4)	0.043
**Average solid component proportion, %**	66.8	24.7	<0.001

SD, standard deviation; SPN, Solid pseudopapillary neoplasm; PD, pancreatoduodenectomy; TP, total pancreatectomy; DP, distal pancreatectomy; CP, central pancreatectomy.

### Imaging Data

The tumor size for the overall study cohort was 47.98 ± 27.75 mm, with a significantly larger tumor size in female patients (50.38 vs 39.65 mm, P=0.038). In the male group, 23/5/4/21 tumors were located in multiple head/body/tail sites of the pancreas, while 67/19/19/63 tumors were located in multiple head/body/tail sites of the pancreas. The tumor location was not different between the 2 groups (χ^2 =^ 0.482, P=0.92).

All patients underwent preoperative imaging evaluations. The imaging diagnoses were correct, which means that the diagnosis was SPN, which was observed in 146 patients (66.9%). In the male group, 28 patients (54.0%) had correct imaging diagnoses. In the female group, 118 patients (70.5%) had correct imaging diagnoses. SPNs in male patients tended to be misdiagnosed with malignant tumors more often than SPNs in female patients (37.7% vs 10.7%, χ^2 =^ 20.662, P<0.0001). The diagnostic accuracy was significantly higher in the female group (χ^2 =^ 5.446, P=0.02). SPNs in male patients tended to be misdiagnosed with malignant tumors more often than SPNs in female patients (37.7% vs 10.7%, χ^2 =^ 20.662, P<0.0001).

Moreover, 49 male patients (92.5%) were diagnosed with having surgical indications (28 SPNs, 20 malignant tumors, 1 NET). Meanwhile, 147 male patients (87.5%) were diagnosed with having surgical indications (118 SPNs, 18 malignant tumors, 11 NETs). The diagnostic accuracy of having surgical indication was not significantly different between the 2 groups (χ^2 =^ 0.415, P=0.519). However, SPNs in male patients tended to be misdiagnosed with malignant tumors more often than SPNs in female patients (37.7% vs 10.7%, χ^2 =^ 20.662, P<0.0001).

Completely cystic tumors were observed in 0 and 32 (19.0%) male and female patients, respectively. Completely solid tumors were observed in 25 (47.2%) and 40 (23.8%) male and female patients, respectively. Tumors that had a solid component <50% were observed in 5 (9.4%) and 41 (24.4%) males and females, respectively. Tumors that had a solid component >50% were observed in 23 (43.4%) and 55 (32.7%) males and females, respectively. The mean solid component was 66.8% ± 24.5 for male patients and 24.7% ± 20.5 for female patients, which was significantly higher in male patients (t=23.67, P<0.0001). The imaging data of the study cohort are shown in **Table 1**.

### Pathological Outcomes

According to the pathological specimens in male patients, a positive margin status was observed in 3 patients (5.7%), and peripheral tissue invasion was observed in 4 patients (7.5%)

For immunohistochemical staining, beta catenin was expressed in 47 cases (89.2%), cyclin D1 was expressed in 50 cases (94.6%), LEF1 was expressed in 33 cases (62.3%), and vimentin was expressed in 44 cases (83.0%). Grade I Ki67 was identified in 47 tumors (88.7%), Grade II Ki67 was identified in 4 tumors (7.5%) and Grade III Ki67 was identified in 2 tumors (3.8%).

In female patients, positive margin status was observed in 14 patients (8.3%), and peripheral tissue invasion was observed in 11 patients (6.5%). For immunohistochemical staining, beta catenin was expressed in 165 cases (98.2%), cyclin D1 was expressed in 164 cases (97.6%), LEF1 was expressed in 105 cases (62.5%), and vimentin was expressed in 108 cases (64.3%). Grade I Ki67 was identified in 141 tumors (83.9%), Grade II Ki67 was identified in 25 tumors (14.9%) and Grade III Ki67 was identified in 2 tumors (1.2%).

Comparing the pathological outcomes between the 2 groups. Positive margin status and peripheral tissue invasion were not different between the 2 groups (χ^2 =^ 0.405 and 0.564, P=0.524 and 0.489). The expression of Cyclin D1 and LEF1 was not significantly different between the 2 groups (χ2 = 0.463, 0.059, P=0.541, 0.808). The Ki67 grade was not different between the 2 groups (χ2 = 1.263, P=0.532). However, the expression of beta catenin was significantly lower in male patients (χ2 = 9.377, P=0.002), and the expression of vimentin was significantly higher in male patients (χ2 = 6.584, P=0.01). The pathological outcomes of the study cohort are shown in **Table 2**.

**Table 2:** Pathological outcomes stratified by gender.

	Male (N=53)	Female (N=168)	*P* value
**Positive margin status, N%**	3(5.7)	14(8.3)	0.524
**peripheral tissue invasion, N%**	4(7.5)	11(6.5)	0.489
**Immunohistochemical staining, N%**			
Beta Catenin	47(88.7)	165(98.2)	0.002
Cyclin D1	50(94.3)	164(97.6)	0.541
LEF1	33(62.3)	105(62.5)	0.808
Vimentin	44(83.0)	118(64.3)	0.01
**KI 67 grade, N%**			0.532
Grade I	47(88.7)	141(83.9)	
Grade II	4(7.5)	25(14.9)	
Grade III	2(3.8)	2(1.2)	

LEF1, Lymphoid enhancer-binding factor.

### Short-Term Complications

In the male group, perioperative complications occurred in 10 patients (18.9%). POPF grade II or above developed in 2 patients (2/10, 20.0%), delayed gastric emptying developed in 2 patients (2/10, 20.0%), abdominal infection developed in 4 patients (4/10, 40.0%), bleeding developed in 1 patient (1/10, 10.0%), and 1 patient (1/10, 10.0%) developed both delayed gastric emptying and abdominal infection. Three complications (3/10, 30%) scored Clavien Dindo grade III or above and were considered severe complications.

In the female group, perioperative complications occurred in 30 patients (17.9%). Five patients (5/30, 16.7%) developed severe POPF, 6 patients (6/30, 20.0%) developed delayed gastric emptying, 5 patients developed abdominal infection (5/30, 16.7%), 9 patients developed bleeding (9/30, 3%), and 5 patients (5/30, 16.7%) developed severe POPF, abdominal infection and bleeding. Ten complications (10/30, 33.3%) scored Clavien Dindo grade III or above and were considered severe complications.

The overall perioperative complication rate and severe complication rate were comparable between the groups (χ2 = 0.028 and 0.00, P=0.868 and 1.0). The short-term complications of the study cohort are presented in **Table 3**.

**Table 3:** Short-term complications and long-term follow-up data stratified by gender.

	Male (N = 53)	Female (N = 168)	*P* value
**Short-term complications**			
**POPF grade II or above, N%**	2(3.8)	5(3.0)	0.773
**Delayed gastric emptying, N%**	2(3.8)	6(3.6)	0.945
**Abdominal infection, N%**	4(7.7)	5(3.0)	0.142
**Bleeding, N%**	1(1.9)	9(5.4)	0.289
**Two or more complications occurred, N%**	1(1.9)	5(3.0)	0.67
**Claviene Dindo grade III or above, N%**	3(5.7)	10(6.0)	1.00
**Long-term follow up data**			
**Alimentary stricture, N%**	1(1.9)	1(0.6)	0.423
**Pancreatic endocrine insufficiency, N%**	3(5.7)	5(3.0)	0.362
**pancreatic exocrine insufficiency, N%**	11(20.8)	31(18.5)	0.710
**Both endocrine and exocrine**	2(3.8)	10(6.0)	0.542
**insufficiency, N% Death, N%**	0(0)	2(1.2)	1.00
**Tumor recurrence, N%**	3(5.7)	7(4.2)	0.648

POPF, postoperative pancreatic fistula.

### Long-Term Follow-Up Data

In the male group, alimentary strictures were observed in 1 patient (1.9%). Three patients (5.7%) experienced pancreatic endocrine insufficiency, and 11 patients (20.8%) experienced exocrine insufficiency. Two patients (3.8%) experienced both pancreatic exocrine insufficiency and endocrine insufficiency. In the female group, alimentary strictures were observed in 1 patient (0.6%). Five patients (3.0%) experienced pancreatic endocrine insufficiency, and 31 patients (18.5%) experienced exocrine insufficiency. Ten patients (6.0%) experienced both pancreatic exocrine insufficiency and endocrine insufficiency. The incidence rates of long-term complications were not different between the 2 groups (χ2 = 0.002, P=0.963).

**Figure 2:**
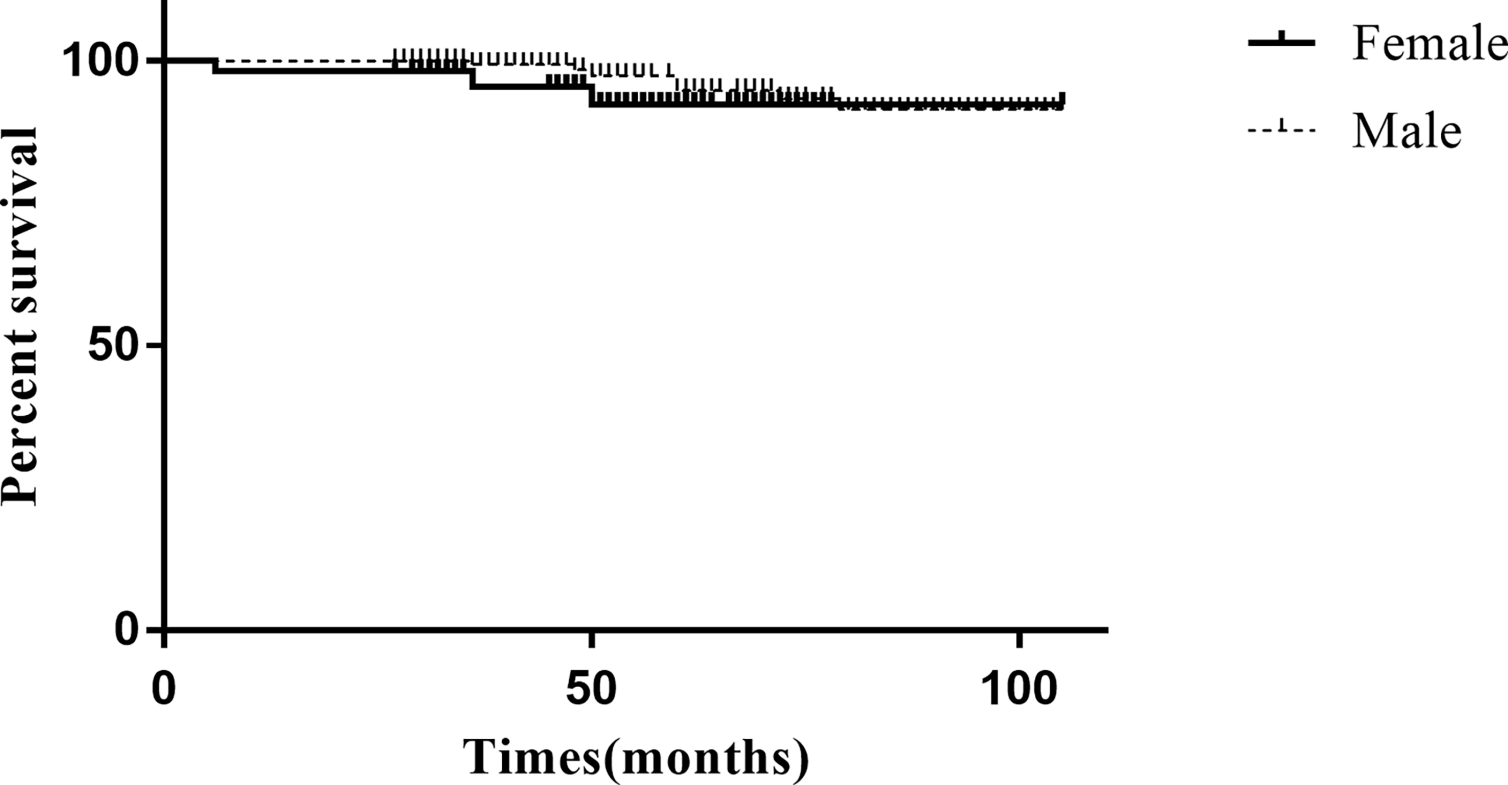
Kaplan–Meier curves for recurrence rate according to the sex.

The final follow-up date was September 30, 2021. The median follow-up period was 54 months (27-105 months). Only 2 patients died due to perioperative complications (both in the female group), and the 3-, 5-, and 8-year overall survival (OS) rates were estimated to be 99.1%, 99.1%, and 99.1%, respectively. In total, 10 patients (4.5%) developed recurrence in the overall study cohort, with 7 patients (5 local recurrence and 2 liver metastasis) in the female group and 3 patients (2 local recurrence and 1 liver metastasis) in the male group. The median time to recurrence was 50 months (range 6–76 months). The 3-, 5-, and 8-year RFS rates were estimated at 98.6%, 96.2%, and 95.3% for the study cohort, respectively. The 3-, 5-, and 8-year RFS rates for the male group were 96.2%, 94.3%, and 94.3%, respectively. The 3-, 5-, and 8-year RFS rates for the female group were 99.4%, 97.0%, and 95.8%, respectively. Kaplan–Meier analysis and the log-rank test showed that the recurrence rate was not significantly different between the groups (χ2 = 1.286, P=0.206) (**Figure 2**). The long-term follow-up data of the study cohort are presented in **Table 3**.

### The Risk Factors for Tumor Recurrence

The parameters, including age, symptoms, surgical method, tumor size, tumor location, margin status, peripheral tissue invasion and immunohistochemical staining, were included in univariate analysis (**Table 4**). The results demonstrated that older age (42.4 vs 32.9 years, P=0.05), large tumor size (67.9 vs 47.1 mm, P=0.04) and a high KI 67 index were significantly associated with tumor recurrence, and the three parameters were included in multivariate logistic regression analysis. Moreover, sex was also included in multivariate analysis to adjust the baseline features.

**Table 4:** Univariate analysis for predication of tumor recurrence.

	Recurrence (N = 10)	No recurrence (N = 211)	*P* value
**Age(years), mean ±SD**	42.4±14.7	32.9±12.5	0.050
**Symptoms, N%**			
incidentally found	5(50)	153(72.5)	0.123
abdominal pain	4(40)	36(17.1)	0.155
abdominal distension	0(0)	12(5.7)	1.00
nausea and vomiting	0(0)	8(3.8)	1.00
jaundice	1(10)	2(0.9)	0.13
**Surgical methods, N%**			
PD	3(30)	58(27.5)	1.00
DP	4(40)	103(48.8)	0.586
CP	1(10)	37(17.5)	0.537
TP	0(0)	10(4.7)	1.00
enucleation	2(20)	11(5.2)	0.210
**Tumor size (mm), mean ±SD**	67.9±35.0	47.1±27.2	0.041
**Tumor location (**head/body/tail/ diffuse**)， N%**	4/0/1/5	86/24/22/79	0.491
**Positive margin status, N%**	1(10)	2(0.9)	0.13
**peripheral tissue invasion, N%**	1(10)	3(1.4)	0.17
**Immunohistochemical staining, N%**			
Beta Catenin	9(90)	203(96.2)	0.332
Cyclin D1	10(100)	204(96.7)	0.808
LEF1	6(60)	132(62.6)	0.786
Vimentin	7(70)	155(73.5)	0.758
**KI 67 grade, N%**			<0.001
Grade I (n=188)	3	185	
Grade II (n=29)	4	25	
Grade III (n=4)	3	1	

Based on multivariate analysis, older age [hazard ratio (HR)= 1.094, 95% confidence interval (CI): 1.005-1.190, P=0.039] and KI 67 index grade III (HR=12.029, 95% CI: 2.399-60.311, P=0.002) were independent risk factors for tumor recurrence (**Table 5**).

**Table 5:** Multivariate analysis for predication of tumor recurrence.

	HR (95%CI)	*P* value
**Age**	1.094(1.005-1.190)	0.039
**Gender**	1.593(0.258-5.678)	0.22
**Tumor size**	1.015(0.973-1.059)	0.484
**KI 67 grade**		
Grade I	1.0	
Grade II	2.589(0.202-33.196)	0.465
Grade III	12.029(2.399-60.311)	0.002

## Discussion

In this study, we analyzed the data of 221 patients with SPN. The primary aim of the study was to identify sex features of SPNs. The second aim of the study was to identify the biological behaviors of SPNs and the risk factors associated with tumor recurrence. Our multiple retrospective study with a large sample size could significantly gain knowledge of SPN.

SPNs can occur at any age but are mainly observed in young women in their 30s (22). In our study, 168 patients were women with a mean age of 31.6 years. However, about one fourth of the patients were male. Data on the characteristics of male patients are scarce. In our study, we identified that male patients were associated with older age, smaller tumor size and more solid components on imaging. The results were consistent with previous studies (14, 23). Beyond these, we observed that male SPNs were previously misdiagnosed by radiologists. SPNs in male patients tended to be misdiagnosed with malignant tumors. However, imaging diagnoses greatly influence the treatment modality and operation type choices. Therefore, the differential diagnosis of SPN for male patients should receive more attention from radiologists. The application of endoscopic ultrasound (EUS) maybe a better solution. Preoperative EUS and EUS guided tissue acquisition allowed pancreatic SPN diagnosis in 80%-90 cases (24, 25). Moreover, the Ki67 index can be measured by the specimens obtained by EUS guided tissue biopsy (26). Hence, in patients whose imaging diagnoses are indeterminate, EUS should be performed.

The difference in male and female SPN may be due to sex hormones (27, 28). Progesterone may participate in the pathogenesis of SPN (29). Some cases reported that SPN grew rapidly during pregnancy (30, 31). Moreover, the estrogen receptor was strongly expressed in tumor tissues. The proliferative action of estrogen *in vitro* was also identified (32). Therefore, the sex features of SPN may be due to exposure to progesterone and/or estrogen during the reproductive period in females (7).

Surgical resection is the standard treatment choice for SPNs. The overall prognosis is excellent for SPNs, with a cure rate of > 95% following complete surgical resection (1, 33). In our study, we also found that the prognosis of SPN was favorable. After surgery, the 3-, 5-, and 8-year OS rates were estimated to be 99.1%, 99.1%, and 99.1%, and the 3-, 5-, and 8-year RFS rates were estimated to be 98.6%, 96.2%, and 95.3%, respectively. At the time of resection, only 7.5% of the patients had peripheral tissue invasion. After long-term follow-up, only 2 patients died due to perioperative complications, and 10 patients experienced tumor recurrence. DFS and OS differences between males and females were not observed in our study. This conclusion was consistent with the study by Cai et al. (16) with 16 cases included. However, Wu et al. (7) and Huffman et al. (17) concluded that female sex was associated with improved survival in which the data were obtained from the National Cancer Center database. The reason may be that the baseline characteristics of the included patients were different from those in our study. In their study, most of their data were obtained from white and black patients. Their sex hormone levels were different from those of Asian patients. Moreover, the average age of male patients was much older than that in our study, and older age was identified as an independent risk factor for tumor recurrence in our study. Therefore, the selection bias of the included patients may contribute to the difference.

The pathological outcomes were also analyzed in our study. More than 90% of the patients could reach margin-negative surgical resection. Peripheral tissue invasion was rare observed. Some previous studies concluded that lymph node metastasis and positive margin status may be risk factors for poor prognosis of SPN (34, 35). However, this relationship was not identified in our study. The inconsistent conclusion may be due to the small number of lymph node metastasis and positive margin status patients included in our study, and statistical bias may exist. Although numerous biomarkers over the years have been documented to have value in diagnosing

SPNs (36–38), there are still no specific immunohistochemical biomarkers for SPNs at present. Only the biomarkers tested in our center were included in our study. The Cyclin D1 expressed in almost all SPNs. Beta Catenin was more specific in female patients, and Vimentin was more specific in male patients. The results may be especially helpful in building pathological diagnoses in tissues obtained by endoscopic ultrasound-guided fine needle biopsy (EUS-FNB). The dose obtained by EUS-FNB tissue is always not as high as that obtained by surgical specimens. However, the conclusion needs further verified.

Ki67 indicates the proliferation of cells. The Ki-67 index has prognostic significance for SPN patients (9, 39). In our study, 3 of the 4 patients who had a grade III Ki67 index experienced tumor recurrence. The recurrence HR for grade III Ki 67 index was 12.03. SPN patients with a grade III Ki67 index were at very high risk of tumor recurrence. A continuous surveillance approach should be adopted in these patients. The cutoff level of the Ki-67 index in our study was based on the criteria for pNETs. For SPT, the optimal cutoff level of Ki-67 needs recalculation. In the studies by Yang et al. (39) and Wu et al. (40), a Ki-67 index greater than 4% was set as the cutoff level. However, only 3 and 4 patients in their studies developed tumor recurrence. Significant statistical bias might exist in their studies. Therefore, we did not adopt the criteria in our study. Due to the small number of cases of SPN, the optimal cutoff level of the Ki-67 index may be difficult to determine due to the small recurrence and death rate.

Patients who had distant metastasis were not included in our study due to the lack of surgical indications. Unresectable SPN remains the most important predictor of poor prognosis in all experiences (41). Nonsurgical treatments have been scarcely investigated, and no standardized protocol exists for this subset of patients. Chemotherapy agents such as cisplatin, 5-fluorouracil and gemcitabine have been proposed in some cases with uncertain results (42). Recently, radiotherapy and targeted therapy have also been reported to treat unresectable or recurrent SPNs in case reports (43, 44). Based on the conclusions in our study, investigating the possible estrogen-dependent behavior of SPN could perhaps open the way to new nonsurgical treatment strategies.

Our study had several limitations worth discussing. First, its retrospective nature prevented us from making stronger conclusions. The second limitation was the relatively small sample size. Due to the rarity of SPNs, SPN cases, especially recurrent SPNs, were not common in our center. More multicenter prospective studies with large sample sizes are necessary to better understand SPNs.

## Conclusion

The incidence of SPN in males was not as low as that previously reported. Male SPN patients were associated with older age, smaller tumor size and more solid components on imaging. For immunohistochemical staining, Cyclin D1 was expressed in almost all SPNs. Beta Catenin was more specific in female patients, and Vimentin was more specific in male patients. Positive margin status, peripheral tissue invasion, postoperative complications, DFS and OS were not significantly different between males and females, which indicated that the prognoses of SPNs in males and females were similar. Elderly age and Ki67 index grade III were independent risk factors for tumor recurrence. Further prospective studies with large sample sizes are needed to verify the findings of our study.

## Data Availability Statement

The raw data supporting the conclusions of this article will be made available by the authors, without undue reservation.

## Ethics Statement

The studies involving human participants were reviewed and approved by The Institutional Review Board of Changhai Hospital. The patients provided their written informed consent to participate in this study.

## Author Contributions

GW, QL, YL, and LS conceived and designed the experiments. YL have made data acquisition. JF, XL, and YS analyzed the data.GW, QL, and LS wrote the manuscript. All authors contributed to the article and approved the submitted version.

## Conflict of Interest

The authors declare that the research was conducted in the absence of any commercial or financial relationships that could be construed as a potential conflict of interest.

## Publisher’s Note

All claims expressed in this article are solely those of the authors and do not necessarily represent those of their affiliated organizations, or those of the publisher, the editors and the reviewers. Any product that may be evaluated in this article, or claim that may be made by its manufacturer, is not guaranteed or endorsed by the publisher.
